# Advances in Complement Inhibitory Strategies for the Treatment of Glomerular Disease: A Rapidly Evolving Field

**DOI:** 10.3390/jcm14124204

**Published:** 2025-06-13

**Authors:** Ester Conversano, Marina Vivarelli

**Affiliations:** 1 Division of Nephrology, Bambino Gesù Children’s Hospital IRCCS, 00165 Rome, Italy; esterconversano@gmail.com; 2 Laboratory of Nephrology and Clinical Trial Center, Bambino Gesù Children’s Hospital IRCCS, 00165 Rome, Italy

**Keywords:** atypical hemolytic uremic syndrome, IgA nephropathy, C3 glomerulopathy, complement system dysregulation, complement inhibition, chronic glomerulopathy

## Abstract

There is rapidly increasing evidence of the role of complement in different forms of kidney disease and this has broadened the field to involve not only atypical hemolytic uremic syndrome (aHUS) and C3 glomerulopathy (C3G), but also a number of other glomerular diseases, mainly ANCA-associated renal vasculitis, immune-complex glomerulonephritis, membranous nephropathy, and IgA nephropathy (IgAN). In parallel, the field of therapeutic agents able to target the three complement pathways at different levels, both proximally and terminally, has grown tremendously in recent years. This has led to the approval of agents targeting complement for ANCA-associated vasculitis, IgA nephropathy, and, very recently, C3 glomerulopathy. The real-world implementation of these agents remains a challenge. This review will attempt, through the presentation of representative clinical vignettes, to provide some practical guidance for the nephrologist in how to navigate these new therapeutic opportunities, focusing on aHUS, C3G, and IgAN.

## 1. Introduction

Complement system dysregulation plays a central pathogenic role in several kidney diseases, particularly atypical hemolytic uremic syndrome (aHUS) and C3 glomerulopathy (C3G). While these conditions differ clinically and histologically, they share the common feature of inappropriate activation of the complement alternative pathway cascade [1]. Recent evidence has shown that complement dysregulation also contributes to inflammation and glomerular damage in IgA nephropathy (IgAN), particularly due to the dysregulation of the lectin and alternative pathways [2].

In aHUS, pathogenic variants in genes encoding regulators of the alternative pathway (such as CFH, CFI, and MCP) or autoantibodies against complement regulatory proteins lead to uncontrolled complement activation at the endothelial surface. Complement blockade with C5 inhibitors has transformed the management of aHUS, preventing progression to kidney failure [3,4].

C3G is characterized by dominant glomerular C3 deposition with minimal immunoglobulin presence, reflecting persistent alternative pathway activation, due to genetic mutations or, more frequently, acquired autoantibodies. Clinical presentation is heterogenous, from mild urinary abnormalities to full-blown nephrotic syndrome with, at times, impaired kidney function. Historically, ~30% of patients develop CKD within 10 years, and post-transplant recurrence exceeds 50% at 5 years. However, these data precede the advent of complement inhibitors, which hold promise in altering disease trajectory [5,6].

In IgAN, the disease driver is the formation of galactose-deficient IgA1-containing immune complexes, which deposit in the mesangium and activate the alternative and lectin pathways. C3 deposition on kidney biopsy is common and correlates with disease severity. Although not yet standard of care, complement inhibition is being explored in pediatric IgAN, particularly in children with high-risk histologic or clinical features [7]. Recently, following the results of a positive randomized controlled trial, iptacopan, a Factor B inhibitor, was FDA-approved for adults with IgAN at risk of rapid progression [8,9].

This review summarizes the current evidence supporting complement inhibition in aHUS, C3G, and IgAN using a series of clinical scenarios, emphasizing pathway-specific targets and therapeutic challenges unique to the pediatric population.

*Case 1. Genetically Proven aHUS: Not Always a Severe Course*

A 12-year-old girl presented with hemolytic uremic syndrome (HUS) characterized by gastrointestinal symptoms, thrombocytopenia, elevated lactate dehydrogenase (LDH), undetectable haptoglobin, moderate anemia, mildly decreased C3 levels, and acute kidney injury (AKI), requiring temporary hemodialysis for 5 days. Renal function subsequently recovered and normalized completely, with supportive therapy alone, within 3–4 weeks.

Given the absence of Shiga toxin-producing infections, the lack of familial history of gastroenteritis, and the atypical age of onset, genetic testing was performed searching for pathogenic variants in complement alternative pathway (AP)-related genes. The patient was found to carry a missense variant in the C3 gene, also present in her father. This variant has been reported as pathogenic for atypical HUS (aHUS).

This case raises a relevant therapeutic dilemma: should all patients with genetically confirmed aHUS receive C5 inhibition? Pathogenic variants in C3 are well-documented to cause severe forms of aHUS, often associated with high recurrence rates after kidney transplantation [10,11,12]. However, clinical expression is variable, and some rare cases remit spontaneously. In this child, full remission was obtained without C5 inhibition, and therefore the genetic panel result created a diagnostic dilemma: should we start treatment in the absence of active aHUS in a child with a completely normal phenotype?

To assess the subclinical degree of complement activation, we employed the HMEC assay, an in vitro test using human microvascular endothelial cells which is a functional tool to assess the individual’s potential for aHUS relapse. The patient’s serum is incubated with HMECs under both resting and activating conditions. The assay measures C5b-9 (membrane attack complex) deposition on the HMEC surface, simulating the terminal complement activation on renal microvasculature in active thrombotic microangiopathy [13,14].

In this patient, the HMEC test was negative in resting conditions and only weakly positive upon activation, suggesting no underlying active complement dysregulation and a limited, albeit present, risk of relapse. Based on this and on the absence of persistent hemolysis, the recovery of kidney function, and normal blood pressure, we opted for clinical and laboratory surveillance, rather than initiating eculizumab. The family was instructed to perform urinary dipstick at least weekly, regular blood exams, and to upscale the monitoring in the event of potential triggers, such as infections or future pregnancy, which may precipitate disease recurrence. During >4 years of follow-up, we have observed no relapse off-therapy.

*Case 2. Genetically Proven aHUS: When to Discontinue C5 Inhibition?*

A 2-year-old boy presented with hemolytic uremic syndrome (HUS) with acute anuric kidney failure requiring dialysis. Kidney biopsy revealed thrombotic microangiopathy (TMA), consistent with the diagnosis. In light of negative microbiological testing, eculizumab therapy was initiated, leading to complete clinical remission and the normalization of renal function. Serial CH50 testing confirmed effective terminal complement inhibition. Genetic analysis revealed a homozygous pathogenic variant in the *MCP* gene. After 9 years of stable remission, a follow-up kidney biopsy showed normal renal parenchyma, with no residual TMA lesions. Based on the long-term remission and histological findings, eculizumab was discontinued. However, two years later, during a family cluster of gastroenteritis, the patient experienced a relapse, presenting with acute kidney injury and signs of early hemolysis. Eculizumab was promptly reintroduced, leading to the rapid recovery of renal function. Upon reinitiating eculizumab therapy, it was necessary to boost vaccinations against encapsulated pathogens. In the meantime, antibiotic prophylaxis was started to reduce the risk of invasive infections.

The discontinuation of anti-complement therapy in aHUS remains a matter of debate, especially in patients with complement gene mutations in MCP, which are traditionally associated with a high recurrence risk, but a milder phenotype, compared to CFH or CFI mutations. The decision to interrupt is easier in patients with an ample renal functional reserve and a kidney biopsy showing little chronic damage. Relapses can occur especially in the setting of triggering events like infections. This case underscores the importance of the rapid detection of signs of relapse and the need for a prompt resumption of C5 inhibition [12].

*How to Protect Patients on C5 Inhibitory Treatment from Infections?*

Patients receiving complement inhibitors are at increased risk of infections caused by encapsulated organisms, such as Neisseria meningitidis, Streptococcus pneumoniae, and Haemophilus influenzae. The recommended immunization schedule includes meningococcal vaccines (both MenACWY and MenB), pneumococcal vaccines, and the Haemophilus influenzae type B vaccine [1,15].

Ideally, the full vaccination schedule should be completed at least two weeks prior to initiating complement inhibitor therapy. If this is not feasible, antibiotic prophylaxis is recommended until two weeks after the last vaccine dose has been administered.

*Case 3. When Complement AP Dysregulation Crosses the Line: C3 Glomerulopathy Meets aHUS*

A 6-month-old infant was admitted with new-onset hemolytic uremic syndrome (HUS), characterized by hemolytic anemia (hemoglobin: 6.5 g/dL), thrombocytopenia (PLT/mm^3^), and laboratory evidence of hemolysis (LDH 2000 U/L, undetectable haptoglobin and the presence of schistocytes on peripheral blood smear). Serum creatinine rose up to 1.4 mg/dL (glomerular filtration rate: 25 mL/min). Additional findings included hypoproteinemia, mild hypocomplementemia (C3 0.7 mg/dL; normal values: 0.9–1.8 mg/dL), hematuria, hypertension, oliguria, and periorbital and lower limb edema. The patient did not present gastrointestinal symptoms. Acute kidney injury (AKI) was managed conservatively without the need for renal replacement treatment.

Negative microbiological investigations supported the diagnosis of atypical hemolytic uremic syndrome (aHUS). Methylmalonic acidemia with homocystinuria and ADAMTS13 deficiency were ruled out. Due to the persistence of hemolysis and acute kidney injury (AKI) by day 6 of hospitalization, treatment with eculizumab was initiated, resulting in rapid clinical improvement.

On day 12, a renal biopsy was performed due to persistent hypoalbuminemia and overt nephrotic-range proteinuria. The histopathological findings were consistent with HUS and concurrent C3 glomerulopathy (C3G), showing glomerular capillary thrombosis along with mesangial, endocapillary, and extracapillary proliferation. Immunofluorescence was positive for C3 deposits.

Genetic analysis identified a de novo pathogenic heterozygous variant in the *CFB* gene. The patient was treated with intravenous steroid pulses followed by oral corticosteroids for six months. Eculizumab infusions were continued due to the high risk of recurrence associated with the identified variants. During follow-up, serum C3 levels remained mildly decreased. At two months following eculizumab initiation, proteinuria had resolved. In the subsequent 3 years of follow-up, she was shifted to ravulizumab, kidney function remained normal without proteinuria, and no relapses were observed.

*Early-Onset aHUS: What to Look for?*

In cases of early-onset HUS, particularly when microbiological investigations exclude typical (Shiga toxin-associated) forms, or in the presence of a severe clinical presentation, evaluation for atypical HUS should be promptly undertaken. This includes metabolic screening for methylmalonic aciduria, assessment of ADAMTS13, and genetic testing for variants in the complement regulatory pathway plus DGKE (Diacylglycerol Kinase Epsilon) variants.

Variants in DGKE lead to endothelial dysregulation, promoting a prothrombotic state; it typically presents within the first year of life with aHUS-associated hypertension and nephrotic-range proteinuria; since the complement pathway is not involved, C3 is not consumed and there are no C3 deposits on kidney biopsy; histology shows features of chronic thrombotic microangiopathy, with glomerular hypercellularity and split glomerular basement membranes; on electron microscopy, podocyte damage with or without focal segmental glomerulosclerosis is present, without dense deposits [16].

*How Should We Diagnose and Treat Coexisting aHUS and C3G?*

C3G and aHUS have been regarded as distinct complement-mediated kidney diseases—the former primarily affecting the glomeruli due to fluid-phase complement dysregulation, and the latter being thrombotic and microangiopathic, driven by endothelial complement dysregulation.

Although rare, atypical HUS and C3 glomerulopathy may coexist in patients with complement gene variants, mainly in CFH mutations. In such scenarios, the new onset or worsening of proteinuria following the resolution of acute kidney injury should suggest coexistent C3G. Renal biopsy is recommended to identify C3G and to evaluate chronic lesions to guide long-term management [17,18].

In cases of combined aHUS-C3G, treatment with eculizumab should be initiated promptly, prior to the confirmation of a genetic variant or the detection of anti-factor H antibodies. Additionally, if membranoproliferative glomerulonephritis is observed, the administration of intravenous pulses of methylprednisolone, followed by oral prednisone with a tapering regimen over a six-month period, may be advisable [1,15,19]. As in C3G, treatment with mycophenolate mofetil may also be beneficial; however, in our case, it was not initiated due to the rapid reduction in proteinuria, the young age of the patient, and the increased infectious risk. In our experience of this and other similar cases, response to C5 inhibition is also optimal in terms of the C3G going into remission [18].

*Case 4. Use of Terminal and Proximal Complement Pathway Inhibitors in C3 Glomerulopathy*

A 11-year-old patient presented a C3 glomerulopathy onset with macroscopic hematuria, reduced kidney function, mild hypertension, mild dysproteinemia, a urine protein-to-creatinine ratio (UPCR) of 3.69 mg/mg, and low circulating C3 levels (10 mg/dL; normal values: 80–180) with normal C4 levels. Complement screening revealed no pathogenic variants, nephritic factor tested negative, and the plasma level of soluble C5b-9, a marker of terminal complement pathway activation, was markedly elevated at 2196 ng/mL (normal: <400).

Initial treatment included intravenous corticosteroid pulses, followed by oral steroids for a total duration of six months, combined with mycophenolate mofetil, which resulted in a slow reduction in proteinuria over 15 months. Upon the relapse of proteinuria (UPCR 1.5 mg/mg), a further course of oral corticosteroids was administered, and cyclosporine was initiated.

Given the ongoing need for dual immunosuppression and the opportunity to participate in an early clinical trial of complement inhibitors, at 13 years of age, the patient was enrolled in the CL011_168 trial and began treatment with avacopan. This led to a reduction in proteinuria to approximately UPCR 0.5 mg/mg; however, cyclosporine could not be discontinued due to the recurrence of proteinuria after CNI tapering [20].

After about 5 years, in order to achieve improved and sustained control of proteinuria, the patient interrupted avacopan and commenced treatment with iptacopan. This allowed for the permanent discontinuation of calcineurin inhibitors while maintaining the remission of proteinuria.

*Avacopan—Terminal Complement Pathway Inhibitor*

Avacopan is a terminal complement pathway inhibitor that selectively blocks the C5a receptor (C5aR1), thereby inhibiting the proinflammatory effects of C5a, a powerful anaphylatoxin which recruits macrophages and neutrophils, without interfering with the formation of the membrane attack complex (MAC and C5b-9). This differentiates it from eculizumab, a monoclonal antibody that binds directly to C5, preventing its cleavage into C5a and C5b and thereby blocking MAC formation downstream. A major advantage of avacopan is its more selective anti-inflammatory action, preserving host defense mechanisms that rely on MAC formation. In addition, avacopan is administered orally, whereas eculizumab requires intravenous infusion [5,20].

Avacopan, currently approved for the treatment of ANCA-associated vasculitis, has been tested in other complement-mediated diseases, including C3 glomerulopathy (C3G), where the ACCOLADE study, though showing improvement in proteinuria in some patients, did not meet its primary endpoint of histological improvement [21].

*Iptacopan—Alternative Pathway Inhibitor*

Iptacopan is a small molecule, administrated orally, that exerts its effect by dose-dependently inhibiting complement Factor B (FB), a critical component in the formation and amplification of the C3 convertase (C3bBb) of the alternative pathway. This inhibition blocks the cleavage of C3 into C3a and C3b, the subsequent formation of the C5 convertase, and the downstream generation of the MAC (C5b-9). Importantly, iptacopan preserves the function of the classical and lectin pathways, allowing for residual MAC generation and maintaining a degree of immune defense. It is under investigation both for aHUS and C3G, and has recently obtained FDA approval both for adults with C3G and for adults with IgAN at high risk of progression [5,22].

*Case 5. Old but Gold—Eculizumab in C3G*

A 12-year-old girl presented with an onset of C3G characterized by nephrotic syndrome with hypocomplementemia, mildly reduced renal function (serum creatinine 1.14 mg/dL), and moderate hypoalbuminemia. She was initially treated with three intravenous pulses of methylprednisolone, followed by a 6-month course of oral steroid therapy in combination with mycophenolate mofetil (MMF) and an ACE inhibitor. Genetics for variants in the alternative complement pathway was negative, while testing for C3 nephritic factor (C3NeF) was positive.

After six months, due to persistent nephrotic-range proteinuria, off-label treatment with eculizumab was initiated, resulting in the complete remission of proteinuria, although serum C3 levels remained low. One year after achieving remission, MMF was discontinued. Two years after disease onset, a repeat kidney biopsy showed no active lesions, prompting the discontinuation of eculizumab. This was followed by a relapse of nephrotic-range proteinuria. Eculizumab was reintroduced, leading to a rapid reduction in proteinuria, which stabilized at a UPCR of 0.3–0.4 mg/mg. Given the need for ongoing treatment, the patient was transitioned to the long-acting formulation ravulizumab.

*Efficacy of Eculizumab in C3 Glomerulopathy (C3G)*

Eculizumab is a humanized monoclonal antibody and was the first approved terminal complement inhibitor. It binds to complement component C5, preventing its cleavage by C5 convertases and thus inhibiting the generation of both the proinflammatory C5a and the cytolytic C5b-9 membrane attack complex (MAC) [5].

The efficacy of eculizumab in C3G remains variable across studies. Complete remission has been reported in approximately 30% of treated patients. Treatment appears to be more effective in histologically active forms of the disease (e.g., crescentic lesions, rapidly progressive glomerulonephritis, or the presence of thrombotic microangiopathy) compared to indolent or slowly progressive forms [3,18,23,24].

As the drug effectively blocks terminal complement activation, serum C3 normalization does not occur. Considering that C3G is mainly driven by AP dysregulation, it appears likely that proximal complement inhibition, with, for example, iptacopan, a Factor B inhibitor, or pegcetacoplan, a pegylated C3 inhibitor, may be more effective [5]. The primary adverse events are related to an increased susceptibility to infections by encapsulated bacteria, which can be effectively prevented by initiating antibiotic prophylaxis until the completion of the appropriate vaccinations [1].

*Switching from Eculizumab to Ravulizumab*

Ravulizumab is a long-acting humanized monoclonal antibody targeting the same C5 epitope as eculizumab. It offers the advantage of extended dosing intervals—administered in adults every 8 weeks, compared to every 2 weeks for eculizumab—without compromising complement blockade. Switching from eculizumab to ravulizumab is considered appropriate in clinically stable patients with C3G or aHUS. The transition requires no overlap or washout period. Ravulizumab should be administered on the same day the next eculizumab dose is scheduled [25].

*To Date, What Is the Optimal Treatment Approach for C3G?*

The standard treatment for C3G includes optimal conservative treatment with low sodium diet and RAS (Renin–Angiotensin System) blockade. In addition to this, a 6-month course of oral corticosteroids, with or without intravenous methylprednisolone pulses, depending on the degree of endocapillary and extracapillary proliferation observed on renal biopsy, and mycophenolate mofetil (MMF) should be initiated in cases with intense proteinuria and proliferative glomerulonephritis [1,26].

Recent clinical trials have shown that avacopan, a C5a receptor antagonist, did not demonstrate efficacy in reducing proteinuria compared to placebo in C3G patients [21].

In February 2025, following positive results of a phase III clinical trial (CLNP023B12301, ClinicalTrials.gov ID NCT04817618), iptacopan, an oral Factor B inhibitor, was approved for the treatment of C3G in adults. This trial is currently enrolling pediatric patients aged 12 years and older with C3G.

Additionally, promising results have emerged from the phase II trial of pegcetacoplan, a targeted C3 inhibitor. By blocking C3, pegcetacoplan prevents the activation of all downstream components of the complement cascade, including C5a and the C5b-9 membrane attack complex (MAC). In the phase II study (NCT03453619) pegcetacoplan met its primary endpoint, achieving a significant reduction in proteinuria and a reduction in C3 deposits in the kidney [27]. The NOBLE trial, conducted in post-transplant adult patients with recurrent C3G or IC-MPGN, showed that pegcetacoplan-treated patients presented a reduction in UPCR of 50% and stable estimated glomerular filtration rate (eGFR) [27]. A phase III trial (NCT05067127), including both adult and pediatric patients with C3G and immune-complex membranoproliferative glomerulonephritis, also met its primary endpoint, demonstrating significant proteinuria reduction compared to placebo [28].

In cases of non-response to standard immunosuppressive therapy, patients with C3G should be referred to a specialized nephrology center to assess eligibility for ongoing clinical trials and access to emerging proximal complement inhibitors [1].

*Case 6. When to Think About Complement Inhibitors in IgAN?*

A 14-year-old patient was diagnosed with IgA nephropathy (Oxford score M1, E0, S1, T0, C1), with preserved renal function and UPCR 2.2 mg/mg. Initial treatment with oral prednisone and RAS blockade led to partial remission (UPCR 0.4 mg/mg). After steroid withdrawal, proteinuria remained stable under ramipril alone. At 16 years old, due to a rise in proteinuria, losartan was added. At 17 years old, the patient developed AKI (creatinine: 2.4 mg/dL) during a febrile illness with prolonged gross hematuria. A second biopsy confirmed active IgA nephropathy (M1, E1, S1, T0, C1) with signs of acute tubular injury and positive IgA +++ and C3 ++ staining. Intravenous steroid pulses were administered, followed by maintenance with oral prednisone for 6 months. Given the background of obesity, budesonide (9 mg/day) was initiated as a systemic steroid-sparing strategy. However, following tapering, proteinuria relapsed (UPCR: 0.96 mg/mg). Once the patient reached adulthood, the initiation of iptacopan therapy was considered. Iptacopan was administered at standard dosing [29]. At the six-month follow-up, proteinuria had decreased to <0.5 mg/mg UPCR, with stable renal function. The patient experienced uncomplicated pneumonia requiring hospitalization, rapidly responding to antibiotic treatment.

IgAN is a renal disorder characterized by the deposition of galactose-deficient IgA1 (Gd-IgA1) in the mesangium that leads to the activation particularly of the alternative pathway complement system. Additionally, the lectin pathway, activated by the binding of mannose-binding lectin (MBL) to Gd-IgA1, further amplifies complement activation. However, to date, the only trial evaluating lectin pathway inhibition with narsoplimab was discontinued prematurely due to lack of efficacy (NCT03608033). This process triggers a cascade of inflammatory events accompanied by C3b deposition, leading to glomerular damage. Complement staining has been associated with worse kidney survival in IgAN patients [8,30,31,32].

Among complement inhibitors, iptacopan (LNP023) is the only one approved for the treatment of adult patients with IgA nephropathy. It is an oral Factor B inhibitor, designed to prevent the activation of C3 by inhibiting the cleavage of Factor B in the C3 convertase complex, a crucial step in the activation of the alternative pathway (AP). In the phase III APPLAUSE trial, it resulted in a significant and sustained reduction in proteinuria compared to placebo. Phase II/III trials with terminal (avacopan, ravulizumab, and cemdisiran) and with other proximal complement inhibitors (RO7434656, vemircopan, and ARO-C3) are ongoing. The use of narsoplimab, a lectin pathway blocker, in IgAN, after encouraging results in a phase II study, has been discontinued due to lack of efficacy in a phase III trial. By interrupting the complement cascade, these therapies offer a potential therapeutic avenue for high-risk IgAN patients, particularly those with persistent proteinuria despite conventional treatments. In children, the use of complement inhibition may be especially beneficial, as they often exhibit a more pronounced inflammatory component and less sclerotic damage compared to adults [2].

*Case 7. Should We Consider Complement Inhibitors Also in IgA Vasculitis with Nephritis?*

A 12-year-old patient diagnosed with IgA vasculitis nephritis was treated with RAS blockade and intravenous steroid pulses (Pozzi protocol), and then she received a single rituximab infusion 3 years later due to persistent proteinuria [33]. At 16 years old, she relapsed with cutaneous purpura and worsening proteinuria, prompting the initiation of mycophenolate mofetil. A second kidney biopsy revealed global and segmental glomerulosclerosis and cellular crescents, indicating active and chronic lesions. High-dose intravenous methylprednisolone was administered.

Due to an acute infectious event, mycophenolate was permanently stopped. The patient continued on alternate-day low-dose steroids. Renal function progressively declined, with an estimated GFR of approximately 35 mL/min/1.73 m^2^, indicating moderate-to-severe chronic kidney disease. The spot protein/creatinine ratio was about 1.75–1.5 mg/mg.

Given the poor response to previous immunosuppressive therapies and the deterioration of kidney function despite relatively mild chronic changes in the kidney biopsy, compassionate-use treatment with ravulizumab, a terminal complement inhibitor, was initiated. Standard dosing as recommended for aHUS treatment was administered. At the three-month follow-up, the patient showed improvement in proteinuria (spot protein/creatinine ratio: 0.88 mg/mg) with stable serum creatinine. The patient did not experience side effects.

Although specific clinical trials in IgA vasculitis (IgAV) remain limited, the shared underlying pathogenesis with IgA nephropathy—particularly involving the activation of the alternative and lectin complement cascades—support the rationale for evaluating complement inhibitors as a potential therapeutic strategy in selected, treatment-resistant cases [34]. In IgAN, a recent phase II study on the use of ravulizumab in adults showed a significant reduction in proteinuria and eGFR stabilization compared to placebo. A phase III trial enrolling adults with IgAN at high risk of progression is ongoing (NCT06291376).

## 2. Conclusions

The field of complement inhibition is rapidly expanding, and as our understanding of complement dysregulation in different forms of kidney disease evolves, more therapeutic options targeting this system emerge. Table 1 provides an overview of the current agents being investigated in aHUS, C3G, and IgAN. Many questions remain unanswered, concerning primarily when to start complement inhibition, which agent to choose as head-to-head comparisons are lacking, and when and how to discontinue this therapeutic approach. Caution to avoid serious infectious complications, particularly meningococcal infections with C5 inhibitors, remains of paramount importance. Last but not least, these new therapies come at great cost, creating issues of equity and availability which cannot be forgotten and which obligate the nephrologist to select the most cost-effective therapeutic strategy, balancing the expense of the drug that may prove most effective against the monetary and human cost of living with chronic kidney disease. Moving forward, these challenges will have to be overcome by a concerted effort uniting physicians, patients, regulatory agencies, and health care providers.

## Figures and Tables

**Table 1:** Main complement inhibitors, mechanisms of action, clinical indications, and/or ongoing clinical trials. UPCR, urine protein/creatinine ratio; IV, intravenous.

Drug Name	Target/ Mechanism	Route of Administration	Approval Phase/Indications (Pediatric/Adult)
Eculizumab	C5/monoclonal antibody	IV	**aHUS:** Approved by EMA (2011) in adults and children. **C3G:** Observational studies showed variable clinical responses; approximately 30–40% improvement in proteinuria.
Ravulizumab	C5/monoclonal antibody	IV	**aHUS:** Approved by EMA (2019) in adults and children. **IgAN:** Phase II study in adults completed; 30% reduction in proteinuria,; phase III study recruiting (NCT06291376).
Crovalimab	C5/monoclonal antibody	IV/SC	**aHUS:** Phase III trial on pediatric patients > 28 days and adults, recruiting (NCT04958265, NCT04861259).
Cemdisiran	C5/RNAi	IV/SC	**aHUS:** Phase II clinical trial for switching from eculizumab to cemdisiran in patients aged > 12 years withdrawn. **IgAN:** Phase II trial on adult patients showed approximately 30–40% reduction in proteinuria.
Avacopan	C5aR/small molecule	oral	**C3G:** Phase II trial in patients > 12 yr completed; no significant changes in UPCR and eGFR. **IgAN:** Phase II trial in adults (NCT02384317); recruitment completed. **aHUS:** Phase II trial in adult dialysis patients terminated.
Iptacopan	Factor B/small molecule	oral	**IgAN:** Phase III adult trial, showing significant and sustained reduction in proteinuria, completed. Approved by FDA/EMA (2024) for adults. **C3G:** Phase III trial on patients > 12 yr, showing significant reduction in proteinuria, ongoing; completed for adults. Approved by FDA/EMA (2025) for adults. **aHUS:** Phase III trial on adult patients naïve from complement inhibitors recruiting (NCT04889430).
Ruxoprubart (NM8074)	Factor Bb/monoclonal antibody	IV/SC	**C3G:** Phase I/II adult trial ongoing (NCT05647811).
ARO-C3	C3/RNAi	SC	**C3G:** Phase I/II adult trial ongoing (NCT05083364). **IgAN:** Phase I/II adult trial ongoing; preliminary results: reduction in UPCR of 40% (NCT05083364).
SGB-9768	C3/RNAi	SC	**C3G, IgAN:** Phase II adult trial ongoing (NCT06786338).
Sefaxersen (RO7434656)	Factor B/mRNA	SC	**IgAN:** Phase II adult trial showed reduction in proteinuria and stabilization of GFR. Phase III adult trial ongoing (NCT05797610).
Vemircopan	Factor D/serine protease	oral	**IgAN:** Phase II adult trial; recruitment terminated.
Pegcetacoplan	C3/synthetic peptide, direct inhibitor	SC	**C3G:** Phase II study on adults showed 50% reduction in proteinuria and stable GFR. Phase II study on post-transplant adult patients with recurrent C3G or IC-MPGN showed 50% reduction in UPCR and stable eGFR. Phase III study on patients aged ≥12 years with C3G or IC-MPGN showed significant reduction in proteinuria.
Narsoplimab	MASP-2/monoclonal antibody	IV	**IgAN:** Phase III adult trial did not achieve statistical significance in reducing proteinuria and was terminated.
Zaltenibart (OMS906)	MASP-3/monoclonal antibody	IV	**C3G**, **IgAN:** Phase II adult patients recruiting (NCT06209736).

## Data Availability

Not applicable.
